# Hypothetical interventions on frailty trajectory among older gastric cancer patients using parametric G-formula

**DOI:** 10.1093/geroni/igaf122.1090

**Published:** 2025-12-31

**Authors:** Xinyi Xu, Yinning Guo, Qin Xu

**Affiliations:** Nanjing Medical University, Nanjing, Jiangsu, China (People’s Republic); Nanjing Medical University, Nanjing, Jiangsu, China (People’s Republic); Nanjing Medical University, Nanjing, Jiangsu, China (People’s Republic)

## Abstract

**Background:**

Frailty is prevalent in older gastric cancer patients, seriously affecting prognosis. This study aimed to estimate the effect of hypothetical interventions on frailty heterogeneous trajectories (FHT) to design targeted intervention programs.

**Methods:**

Data from a longitudinal follow-up study of 381 gastric cancer patients ≥60 years were collected at admission, discharge, and 1, 3, 6, and 12 months post-surgery. The parametric g-formula estimated the effects of single and joint hypothetical interventions on FHT risk across physical, psychological, familial, and social factors.

**Results:**

The observed FHT risk was 43.57%. Single intervention on nutrition most effectively reduced FHT risk (RR = 0.790, 95% CI: 0.692-0.974), followed by family cohesion (RR = 0.810) and social objective support (RR = 0.822). The “all-factors” joint intervention reduced risk by 31.26% (RR = 0.323, 95% CI: 0.208-0.685). An evidence-based intervention program was designed with good precision and scientificity.

**Conclusions:**

Based on optimal intervention combinations determined from parametric g-formula, we designed a comprehensive FHT intervention program covering multiple time periods, scenarios and factors, providing theoretical and practical guidance for frailty prevention in older gastric cancer patients.

